# eHealth supported multi-months dispensing of antiretroviral therapy: a mixed-methods preference assessment in rural Lesotho

**DOI:** 10.1186/s40814-022-01019-x

**Published:** 2022-03-11

**Authors:** Ramona Scherrer, Nadine Tschumi, Thabo Ishmael Lejone, Mathebe Kopo, Lipontso Motaboli, Buoang Mothobi, Alain Amstutz, Michael J. Deml, Malebanye Lerotholi, Niklaus Daniel Labhardt

**Affiliations:** 1 Clinical Research Unit, Department Medicine, Swiss Tropical and Public Health Institute, Basel, Switzerland; 2 University of Basel, Basel, Switzerland; 3 SolidarMed, Swiss Organization for Health in Africa, Butha-Buthe, Lesotho; 4 Division of Infectious Diseases and Hospital Epidemiology, University Hospital Basel, Basel, Switzerland; 5 Institute of Sociological Research, Department of Sociology, University of Geneva, Geneva, Switzerland; 6 Division of Social and Behavioural Sciences, School of Public Health & Family Medicine, University of Cape Town, Cape Town, South Africa; 7 Ministry of Health of Lesotho, Maseru, Lesotho

**Keywords:** HIV, Differentiated service delivery (DSD) model, Antiretroviral therapy (ART), People living with HIV (PLWH), Multi-month dispensing (MMD), Viral load (VL), eHealth, Telemedical support

## Abstract

**Background:**

Multi-month dispensing (MMD) of antiretroviral therapy (ART) represents one approach of differentiated service delivery (DSD) aiming to improve quality and cost-effectiveness for HIV services in resource-limited settings. However, reduction in clinic visits for people living with HIV (PLWH) should go along with out-of-clinic care tailored to PLWH`s preferences and comorbidities to maintain quality of care. eHealth supported MMD offers a potential solution.

**Methods:**

Between October 2019 and January 2020 we assessed preferences on an eHealth supported MMD package among adult PLWH attending routine ART care at a rural clinic in Lesotho using a mixed-methods approach. Participants reported their preferences among different refill and eHealth options. They were invited to test automated text messages (SMS) informing about their viral load results, an automated tuberculosis symptoms screening call and telemedical support by an expert nurse. Telemedical service comprised a call-back option if participants required any additional support and adherence counselling for closer follow-up of participants with unsuppressed viral loads. After 6 weeks, participants were followed-up to assess perception of the chosen eHealth support using a qualitative approach.

**Results:**

Among 112 participants (median age = 43 years; 74% female), 83/112 (75%) preferred MMD for 6–12 months (median = 9 months, IQR = [5, 12]). Neither sex, age, employment, costs and time for travel to clinic, nor the duration of taking ART correlated with the MMD preference. All 17 participants attending routine viral load measurement wished to receive the result via SMS. Fifteen (19.2%) participants requested a telemedical nurse call-back during the study period. All participants with recent unsuppressed viral load (*N* = 13) requested telemedical adherence counselling for closer follow-up. Among 78 participants followed-up, 76 (97%) would appreciate having the call-back option in future. Seventy-five participants (67%) received and evaluated the automated symptomatic tuberculosis screening call, overall 71 (95%) appreciated it.

**Conclusions:**

The great majority of PLWH in this study preferred 6–12 months MMD and appreciated the additional eHealth support, including viral load results via SMS, telemedical nurse consultations and automated tuberculosis symptom screening calls. eHealth supported MMD packages appear to be a promising approach for DSD models and should be assessed for clinical endpoints and cost-effectiveness in larger studies.

**Supplementary Information:**

The online version contains supplementary material available at 10.1186/s40814-022-01019-x.

## Key messages regarding feasibility

1. What uncertainties existed regarding the feasibility?

- Preferences regarding multi-month ART dispensing among PLWH in Lesotho
- Uptake, acceptance and feasibility of eHealth support in addition to multi-month ART dispensing among PLWH in Lesotho

2. What are the key feasibility findings?

- The large majority opted for multi-month ART dispensing with refill-intervals of 6 months or longer
- Uptake and acceptance of eHealth support in addition to multi-month ART dispensing was high
- The different eHealth elements piloted appeared feasible and implementable at a larger scale

3. What are the implications of the feasibility findings for the design of the main study?

- The here piloted care model of multi-month ART dispensing combined with eHealth support appears to be a promising and scalable approach to support differentiated service delivery in Lesotho and similar settings in Southern Africa
- The piloted model should be assessed for clinical endpoints and cost-effectiveness in larger-scale studies

## Background

Differentiated service delivery (DSD) models have been proposed to sustainably optimize HIV services along the care cascade in resource-limited settings [1–5]. DSD models aim to transition from a “one-size-fits-all” approach to a model of care tailored to specific clinical and contextual needs of groups of people living with HIV (PLWH). Thereby, it bears the chance to allocate resources more efficiently to ultimately reduce the burden on health systems while improving quality of care [2, 3, 5, 6].

Most DSD models have so far focused on antiretroviral therapy (ART) services for clinically stable PLWH while less evidence exists for DSD models targeting the full continuum of care [1–6]. Viral load (VL) result-informed DSD models represent an auspicious opportunity to ensure cost-effectiveness of viral load monitoring by triaging patients according to their viral loads and required level of care, thereby allowing to save time and resources in stable patients and to focus on patients with unsuppressed VL results [7–9]. The COVID-19 pandemic has led to an additional push towards DSD for PLWH, particularly the approach of longer visit intervals through multi-month dispensing (MMD) of ART, since it became critical to ensure uninterrupted ART supply while reducing unnecessary contact with health clinics [5–16]. Recently, a pragmatic open-label cluster-randomized trial conducted in Malawi and Zambia showed that 6 months MMD was non-inferior to shorter clinic visit intervals in terms of retention in care [17]. However, in the spirit of DSD, reduction in clinic visits should align with patient preference, take into account comorbidities and the individual’s personal needs and demands [18].

One approach is to combine MMD with electronic health (eHealth) support during longer intervals where patients do not attend the clinic. As the access to mobile phones has immensely increased in Sub-Sahara Africa (SSA), eHealth approaches represent a promising strategy to reach vulnerable populations remotely and to facilitate equitable and affordable healthcare to marginalized areas with poor infrastructure, thus strengthening health systems in providing efficient HIV service [19–25]. With regard to HIV programs, eHealth bears the potential to tackle current inefficiencies in viral load monitoring by ensuring that viral load results are followed by appropriate actions [20–22, 25, 26]. However, to warrant patient-centered care, eHealth tools must be designed according to patients’ preferences [6, 27–29].

This formative pilot study aimed to assess preferences and perception among ART experienced PLWH on an eHealth supported package of care, targeting clinically stable PLWH with MMD options and PLWH with unsuppressed viral loads. This care package includes automatically triggered text-messages on VL results, an automated interactive call for remote symptomatic tuberculosis (TB) screening, telemedical support for enhanced adherence counselling (EAC) in case of unsuppressed VL, and the option of requesting a nurse call-back for any additional telemedical support.

## Methods

### Design

This prospective observational study aimed at assessing PLWHs’ preferences for MMD and acceptance of eHealth support in addition to MMD. A mixed-methodology was applied, combining a quantitatively conducted survey with a complementary qualitative component. In accordance with Creswell [2014], quantitative and qualitative data were collected concurrently in a parallel embedded approach, meaning that analysis was done separately and findings were triangulated [30].

The eHealth support of MMD contained the following four elements: (1) communication of the viral load result by SMS, (2) enhanced adherence counselling (EAC) through a nurse by phone in case of unsuppressed viral load, (3) the option to request a call-back by a nurse in case any support is needed, and (4) an automated interactive TB symptom screening call where the patient was asked about the four symptoms of presumptive TB (cough, night sweats, weight loss, loss of appetite) [31]. For each symptom the patient then had to press “1” for “yes” and “0” for “no.”

This study was conducted as formative work for the VITAL trial—a cluster randomized trial assessing an eHealth supported MMD model in rural Lesotho (https://www.vital-lesotho.org; clinicaltrials.gov: NCT04527874).

### Setting and participants

The study was conducted from 31 October 2019 to 16 January 2020 at the nurse-led missionary health clinic St. Paul in the district of Butha-Buthe, North Eastern Lesotho. All PLWH attending the health clinic during the enrolment period were assessed for participation. PLWH were eligible if they were at least 18 years of age, taking ART for at least 6 months, willing to be followed-up at the clinic 1 month later, currently not taking TB treatment and having access to a mobile phone. Convenience sampling, including all eligible and consenting participants was applied rather than a formally calculated sample size. For the qualitative component of the study, all participants who came for follow-up and who tested the eHealth support tools were sampled on a purposive basis. The study protocol was approved by the National Health and Research Ethics Committee of Lesotho (ID 220-2019). All participants provided written informed consent.

### Procedures

At enrolment, all participants completed a preference assessment about their preferred length of MMD intervals and about their preferences on four components of the eHealth support offered during MMD using a quantitative questionnaire. Thereafter, participants attending for VL testing could choose to get the result via SMS. Participants with recent unsuppressed VL results had the option of enhanced adherence counselling (EAC) by phone. All participants were offered the call-back option in case they wished telemedical advice from a nurse. Further, all participants tested the automated interactive TB symptom screening call and were subsequently interviewed about their understanding and perception of the call.

Six weeks after enrolment, participants were recontacted. All participants who could be recontacted successfully followed a quantitative questionnaire assessing their perception of the different eHealth components. A subsample who tested the eHealth tools underwent qualitative, audio-recorded open-end questions over approximately 5–10 min and were asked to explain why they perceived the eHealth support as advantage or disadvantage, respectively. Detailed processes of eHealth testing can be found in the Supplementary Information.

### Quantitative analysis

Categorical variables were described by absolute and relative frequencies and continuous variables by medians and interquartile ranges (IQR). Where reasonable, we stratified for age and gender, and chi2 or Fisher’s exact test was used for comparison of strata. Missing data were excluded from the analysis. All quantitative analyses were run on STATA V.14.0.

### Qualitative analysis

Qualitative data were obtained from audio-recorded interviews in Sesotho through the use of open-ended questions and with the support of an interview guide which contained questions related to PLWH’s preferences and perception on the tested eHealth components. Audio recordings were transcribed and translated verbatim into English. In agreement between the authors RS and MJD, narratives were analysed through the use of open coding and themes were identified, contextualized and interpreted based on the thematic analysis suggested by Braun and Clarke using a theoretical approach [32]. Finally, findings were converged with quantitative results [30, 32, 33].

## Results

### Study population

**Fig. 1:**
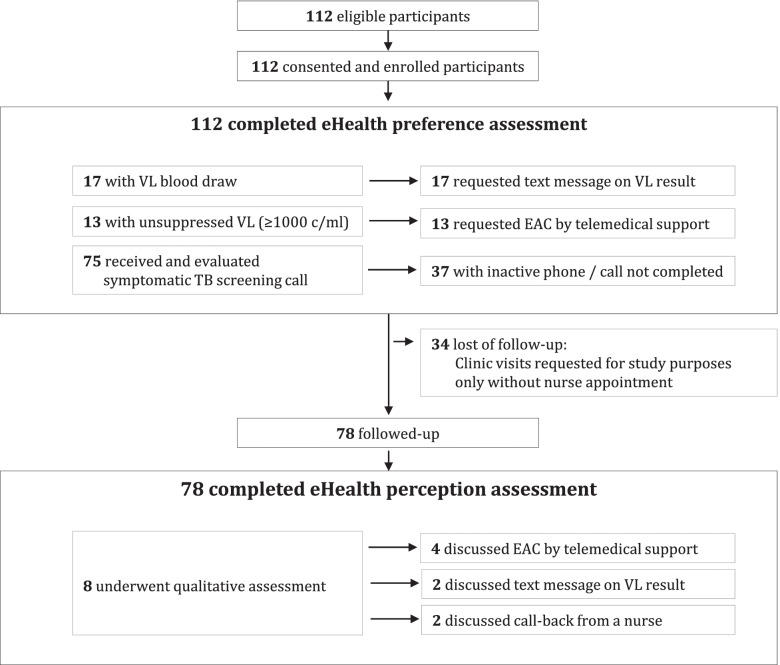
Flow diagram

Figure 1 provides an overview of study participation and follow-up. A total of 112 participants were recruited between October and December 2019 and completed the questionnaire for the eHealth preference assessment. The median age was 43 years, 74% were female, 17 (15.2%) were due for routine viral load monitoring and 13 (11.6%) had a recent last viral load result ≥ 1000 copies/ml. Table 1 displays the participants’ characteristics.

**Table 1:** Participants’ characteristics

***n***	112
**Age (median [IQR])**	43.0 [35.0, 52.5]
**Gender (%)**	
Female	83 (74.1)
Male	26 (23.2)
Unspecified	3 (2.7)
**VL blood draw during enrolment (%)**	
Unknown	1 (0.9)
No	94 (83.9)
Yes	17 (15.2)
**Last VL result ≥ 1000 copies/ml (%)**	
Unknown	2 (1.8)
No	97 (86.6)
Yes	13 (11.6)
**ART regimen (%)**	
2 NRTIs + 1 INSTI	6 (5.4)
2 NRTIs + 1 NNRTI	102 (91.1)
2 NRTIs + 1 PI	4 (3.6)
**Duration of ART (years)** (median [IQR])	4.1 [2.3, 7.6]
**Missed HIV medication in previous month (%)**	16 (14.3)
**Other medication (%)**	
Unknown	1 (0.9)
Co-trimoxazole	1 (0.9)
High blood pressure medicine	32 (28.6)
Combination: high blood pressure medicine and traditional medicine	1 (0.9)
Isoniazid preventive therapy (ITP)	18 (16.1)
No other medication	57 (50.9)
Other	2 (1.8)
**Employment (%)**	
Employed in Lesotho	12 (10.7)
Employed in South Africa	3 (2.7)
Housewife	2 (1.8)
No regular income	86 (76.8)
Self-employed with regular income	8 (7.1)
Subsistence farming	1 (0.9)
**Education (%)**	
Completed high school	12 (10.7)
Completed primary school	38 (33.9)
Completed secondary school	27 (24.1)
Completed tertiary school	4 (3.6)
Primary school not completed	31 (27.7)
**Travel costs to health center** (Maloti) (median [IQR])	8.0 [8.0, 10.0]
**Travel time to health center** (min) (median [IQR])	37.5 [20.0, 45.0]

Of the 112 participants, 75 (67%) received and evaluated the automated TB symptom screening call, the remaining 37 did either not have an active mobile phone or did not complete the call.

At follow-up, 78 (70%) participants could be met in person and interviewed how they perceived the eHealth components they had received during follow-up. Thereof, eight participated in a qualitative interview to discuss their experiences with various aspects of the eHealth options (Fig. 1).

### Quantitative results

#### Preferred clinic visit intervals

The majority (83/112, 75%) preferred MMD ≥ 6 months. Thereof, 26 (31%) opted for 6 months MMD, 13 (16%) for 9 to 10 months MMD and 43 (52%) for 12 months MMD. The median of preferred refill intervals was 9 months (IQR 5–12 months). Neither sex, age, costs and time to travel to the clinic, employment status, nor the time since taking ART correlated with the preferred MMD duration.

#### Automated VL result text messages

For each of the offered SMS options, over 95% of participants stated to opt for it in case it would be offered at their clinic: 109/112, 111/112, and 110/112 wished to receive an SMS informing them in case their viral load result was suppressed, unsuppressed or the viral load measurement needed to be repeated, respectively. Further, 110/112 wished reminder SMS for clinic visits.

All 17 participants, who attended for routine viral load measurement during enrolment opted for receiving their viral load result via SMS.

#### Enhanced adherence counselling by telemedical service

Ranking of different enhanced adherence counselling (EAC) options according to preferences revealed telemedical counselling as the preferred choice (91/112, 81.25%) followed by ART intake reminders via SMS (14/112, 12.5%) and standard counselling at the clinic (7/112, 6.3%). All 13 participants who attended the clinic during enrolment with the last viral load being unsuppressed ranked EAC through telemedical service as their preferred option and wished to test the phone call support during the study. Four participants, who received the nurse phone call could be followed-up and all four participants appreciated the telemedical eHealth support (“it was helpful”).

#### Nurse call-backs

Among the 112 participants, 76 (68%) stated that they would appreciate a call-back option by a nurse in the future. Among the 78 participants followed-up, 15 (19.2%) had dropped a call-back request and the nurse could successfully contact five to provide counselling. All five stated at follow-up having appreciated this telemedical support (“it was helpful”).

#### Automated symptomatic TB screening

Among the 75 participants who received the TB symptom screening call, 72 (96%) indicated it was easy to follow. Correct understanding and recall of the four symptom questions were as follows: 67/75 (94.5%) for cough, 43/75 (58.1%) for night sweats, 38/75 (51.3%) for weight loss, and 2/75 (2.7%) for fever. Only 46 (61.3%) understood that they had to dial “1” for “yes” and “0” for “no” after each symptom question. Seventy-one of the 75 (95%) stated the automated TB call to be a “good idea as it is” and 4 (5%) stated it to be a “good idea but needs improvement”.

### Qualitative results

Following analysis of the eight qualitative interviews, we identified three overarching themes related to PLWHs’ experiences with the three eHealth support components offered. As all eHealth tools were perceived positively among participants, thematic analysis focused on the main themes of “convenience,” “privacy,” and “efficiency” as advantages of eHealth support related to justifications given (Table 2).

**Table 2:** Thematic analysis

Themes	Codes	Data extracts
Convenience	Facilitated health service delivery by using phones	*“I found it to be very better and making things easy by using phones.” (EAC by telemedical service)*
Facilitated health service receipt at home without clinic visits	*“When I talked with the nurse, I found this work being done now to be useful. We will get our services when we are still at home without having to go the health center many times.”* *(EAC by telemedical service)*	
Privacy	Talking about personal problems in private	*“I am very grateful for it, because I was able to talk to my nurse in private and I explained my personal problems.”* *(Call-back by nurse)*
Talking about health problems on the phone	*“I really talked with a nurse over the phone. We had a good discussion and I told him all my health problems.” (EAC by telemedical service)*	
Efficiency	Direct medical support	*“I learned that in order for viral load not to be in a good condition when I talked with the nurse and I was explained to how things relating to it go.” (EAC by telemedical service)*
*“I found it to be very useful because I did not know how the drugs, I was given were making me feel.” (Call-back by nurse)*		
*“I am very thankful that I got an SMS. After getting the message I felt energetic.” (automated VL result text message)*		
*“I found it to be good when I got a message that my result was low.” (automated VL result text message)*		
Direct behavioral support	*“We really talked, and he told me important information and what I had to do.” (EAC by telemedical service)*	
*“The nurse also explained to me …, how I have to behave…” (Call-back by nurse)*		

Convenience of eHealth support generally refers to participants’ experiences of easier access to health services. Participants who received adherence counselling by phone attributed benefits to the simplified health service delivery using telemedicine and the advantage of getting health support at home without being obliged to frequent clinic visits. Thus, participants emphasized the convenience of telemedical counselling as a major advantage of this support option—as a 43-year-old woman noted, whose last VL results were unsuppressed and wished to received EAC by phone:

“When I talked with the nurse I found this work being done now to be useful. We will get our services when we are still at home without having to go the health center many times. We get the services of adhering well to our medication when we are still at home.”

Privacy of eHealth support encompasses participants’ perception of personal or health counselling within a private atmosphere. Participants who used telemedical support at their desired time highlighted the opportunity to talk with a nurse about personal problems and health issues in their preferred environment—especially about a sensitive topic such as HIV treatment.

Efficiency of eHealth support comprises participants` opinion on direct medical or behavioural information and counselling. The possibility of getting immediate medical and behavioural support by phone in situations needed, e.g., when side effects occur or when questions arise about viral load results, was highly appreciated among participants. Moreover, being directly informed about viral load results through text messages was described as personal relief and continuing motivation for participants with suppressed HIV. In summary, participants identified privacy and efficiency as main advantages of the eHealth support options offered in this study, as the following statements demonstrate:

A woman at the age of 43, who is on ART since seven years and had high last VL results, made use of the call-back option and stated: “The good things about this work and that I am very grateful for it, because I was able to talk to my nurse in private and I explained my personal problems. The nurse also explained to me what I have to do, as well as what to do in order for my viral load to be good, how I have to behave and what has to happen. Therefore, thank you.“

A 40-year-old woman talked with a nurse during a call-back and remarked: “I found it to be very useful because I did not know how the drugs I was given were making me feel, so I realized that they were just changed because I was told that my viral load was very high.”

A woman at the age of 32, who received an SMS about her VL results, noted: “I am very thankful that I got an SMS. After getting the message I felt energetic. I am thankful and these interactions with the nurse to continue.”

## Discussion

Beyond the COVID-19 pandemic, alternative and cost-effective service delivery models need to be developed to address key challenges in the current global HIV/AIDS response—namely, long-term engagement in care and virologic suppression among rising numbers of PLWH [1–6, 14, 16, 17, 34]. This formative pilot study conducted at a rural health center in Lesotho assessed preferences among ART experienced PLWH with regard to MMD and additional eHealth support options. Irrespective of demographic, clinical or socio-economic characteristics, the majority of participants preferred MMD with intervals between 6 and 12 months. Interest was high for individualized text messages on viral load results, symptomatic TB screening and telemedical consultation and follow-up. Results from the quantitative survey and the embedded qualitative component were consistent and in the qualitative analysis, efficiency, privacy, and convenience emerged as the main advantages of offered eHealth support options along with MMD.

To our knowledge, there is currently no published study that assessed eHealth support combined with MMD with regard to programmatic outcomes, such as engagement in care, cost effectiveness and virologic suppression. There is lack of evidence for eHealth interventions using SMS for direct communication of viral load results to empower PLWH, reduce unnecessary clinic visits and to ensure timely support for PLWH with unsuppressed VL. Likewise, the potential of telemedical adherence support for PLWH with unsuppressed viral load appears to be largely unexploited [20–23, 25]. Components of patient-centered care such as facilitated patient-provider communication, shared responsibility, and decision-making are among the key elements of eHealth interventions expected to contributing to favorable HIV outcomes [6, 20–23, 28, 29]. In line with other patient’s preference assessments from similar settings, our eHealth supported MMD package revealed encouraged interaction between patients and nurses by telemedical support and stimulated patients’ empowerment through direct SMS communication [25, 27, 35–42]. In busy clinics, there is often neither privacy nor time for patients to bring up problems or concerns during the ART consultations. Participants in our study made use of the call-back option to talk to a nurse when they were in privacy, indicating demand for additional support through health care professionals. However, fidelity among PLWH and nurses need to be strengthened, as only one-third of those participants could be reached successfully. An adequate grade of patient-centeredness will thereby be crucial to balancing out the out-of-clinic workload for the nurses with effective counselling according to patient’s requirements [28]. Better compliance of the remote symptomatic TB screening call could be achieved by technical improvements like increasing the volume of the automated voice, as suggested by participants who did not understand and recall it correctly. These findings need to be considered for a larger-scale implementation study to warrant advantages of the MMD approach supported by eHealth—such as convenience, efficiency and privacy—without leaving anyone behind [28]. Given the great appreciation rate of our eHealth supported MMD model in our study, potential implications are not only auspicious for retention in care over longer visit intervals especially in times of COVID-19, but also for improving VL monitoring efficiency and management of treatment responses to finally achieve better virologic suppression [21, 22, 28, 40, 43–46].

This study has several limitations. First, it was conducted at a single clinic with a relatively small sample-size. Secondly, this study only assessed patient’s preferences and perception of different eHealth options supporting a MMD approach. A larger-scale study with long-term follow-up is needed to determine if in real life these care options are working, remain appreciated over time and result in good clinical outcomes.

## Conclusions

The majority of PLWH in this study preferred 6–12 months MMD. The further offers of our MMD package—viral load result communication by SMS, telemedical support, and an automated symptomatic tuberculosis screening call—were all well perceived. This eHealth supported MMD package appears to be a promising approach to inform a DSD model targeting the full continuum of care in times of and beyond the COVID-19 pandemic and should be assessed for clinical and programmatic outcomes in larger-scale studies. Based on the findings of this formative pilot study, the project *viral load-triggered ART care in Lesotho (VITAL)* (NCT04527874) will assess eHealth supported MMD for clinical endpoints as well as cost effectiveness.

## Supplementary Information

**Additional file 1: Supplementary Information**. Data collection and processes. All eHealth options were implemented in the local language, Sesotho (Supplementary Figures 1 and 2). The VL result text messages containing encrypted information (minimize the risk of HIV status disclosure) were automatically triggered from a password protected online VL database. The automated interactive symptomatic TB screening call was triggered using tablet technology on site during enrolment. According to WHO recommendations, it encompasses requests for dialing 1=yes or 2=no for the presence of each of the symptoms, including coughing, fever, night sweats and weight loss, while the answers rely on self-assessment of the participants [31] For providing EAC support by telemedical service, an ART nurse was provided a list of participants, who came with recent VL ≥1000 copies/ml and who requested additional EAC by phone at their preferred time and day. For testing the nurse call-back, the phone number from the ART nurse was distributed to all participants during enrolment with the invitation to leave a missed phone call for requesting the call-back at any time. **Supplementary Figure 1.** Design of the automated VL result text messages. **Supplementary Figure 2.** Design of the automated symptomatic TB screening call.

## Data Availability

The datasets used and analyzed during the pilot study are available from the corresponding author on request.

## Abbreviations

ART: Antiretroviral therapy
DSD: Differentiated service delivery
EAC: Enhanced adherence counselling
eHealth: Electronic health
HIV: Human Immunodeficiency Virus
MMD: Multi-month dispensing
PLWH: People living with HIV
SMS: Short message service
SSA: Sub-Sahara Africa
TB: Tuberculosis
UNAIDS: United Nations Programme on HIV/AIDS
VL: Viral load
WHO: Word Health Organization
