# Supplementary material for: eHealth supported multi-months dispensing of antiretroviral therapy: a mixed-methods preference assessment in rural Lesotho
Source: Pilot Feasibility Stud. 2022 Mar 11;8:61. doi: 10.1186/s40814-022-01019-x (PMC8913859; doi:10.1186/s40814-022-01019-x)
Supplement: Supplementary file 1 — Additional file 1: Supplementary Information. Data collection and processes. All eHealth options were implemented in the local language, Sesotho (Supplementary Figures 1 and 2). The VL result text messages containing encrypted information (minimize the risk of HIV status disclosure) were automatically triggered from a password protected online VL database. The automated interactive symptomatic TB screening call was triggered using tablet technology on site during enrolment. According to WHO recommendations, it encompasses requests for dialing 1=yes or 2=no for the presence of each of the symptoms, including coughing, fever, night sweats and weight loss, while the answers rely on self-assessment of the participants [31] For providing EAC support by telemedical service, an ART nurse was provided a list of participants, who came with recent VL ≥1000 copies/ml and who requested additional EAC by phone at their preferred time and day. For testing the nurse call-back, the phone number from the ART nurse was distributed to all participants during enrolment with the invitation to leave a missed phone call for requesting the call-back at any time. Supplementary Figure 1. Design of the automated VL result text messages. Supplementary Figure 2. Design of the automated symptomatic TB screening call. [file 40814_2022_1019_MOESM1_ESM.zip › Suppl Fig 1_Automated VL result text messagesR1.pdf]

**VL < 40**	**VL 40-999**	**VL ≥ 1'000**	**Technical failure of VL measurement**
Congratulations on good test result ([result])! Keep it up! Greetings, Saint Paul	*Your test ([result]) needs to be repeated in 3 months. Greetings, Saint Paul*	*Your test result is out, please come and see us as soon as possible. Greetings, Saint Paul*	*Your test should be repeated, sorry for inconvenience. Greetings, Saint Paul*
Rea u lebohela, sepetho sa hao se hantle haholo,Ts'oara joalo. Litumeliso ho tsoa setsing sa St Paul	*Sephetho sa hau se tlameha ho phethoa ka mora khoeli tse tharo.Litumeliso ho tsoa setsing sa St Paul*	*Sephetho sa hau se tsuile, re kopa u itlhalehe setsing kapele hau fumana molaetsa. Litumeliso ho tsoa setsing sa St Paul*	*Sephetho sa hau se tlameha ho phethoa, itlalehe setsing kapele hau fumana molaetsa. Re kopa ts'oarelo ka ts'itiso..Litumeliso ho tsoa setsing sa St Paul*
