# Supplementary material for: eHealth supported multi-months dispensing of antiretroviral therapy: a mixed-methods preference assessment in rural Lesotho
Source: Pilot Feasibility Stud. 2022 Mar 11;8:61. doi: 10.1186/s40814-022-01019-x (PMC8913859; doi:10.1186/s40814-022-01019-x)
Supplement: Supplementary file 1 — Additional file 1: Supplementary Information. Data collection and processes. All eHealth options were implemented in the local language, Sesotho (Supplementary Figures 1 and 2). The VL result text messages containing encrypted information (minimize the risk of HIV status disclosure) were automatically triggered from a password protected online VL database. The automated interactive symptomatic TB screening call was triggered using tablet technology on site during enrolment. According to WHO recommendations, it encompasses requests for dialing 1=yes or 2=no for the presence of each of the symptoms, including coughing, fever, night sweats and weight loss, while the answers rely on self-assessment of the participants [31] For providing EAC support by telemedical service, an ART nurse was provided a list of participants, who came with recent VL ≥1000 copies/ml and who requested additional EAC by phone at their preferred time and day. For testing the nurse call-back, the phone number from the ART nurse was distributed to all participants during enrolment with the invitation to leave a missed phone call for requesting the call-back at any time. Supplementary Figure 1. Design of the automated VL result text messages. Supplementary Figure 2. Design of the automated symptomatic TB screening call. [file 40814_2022_1019_MOESM1_ESM.zip › Suppl Fig 2_Automated symptomatic TB screening callR1.pdf]

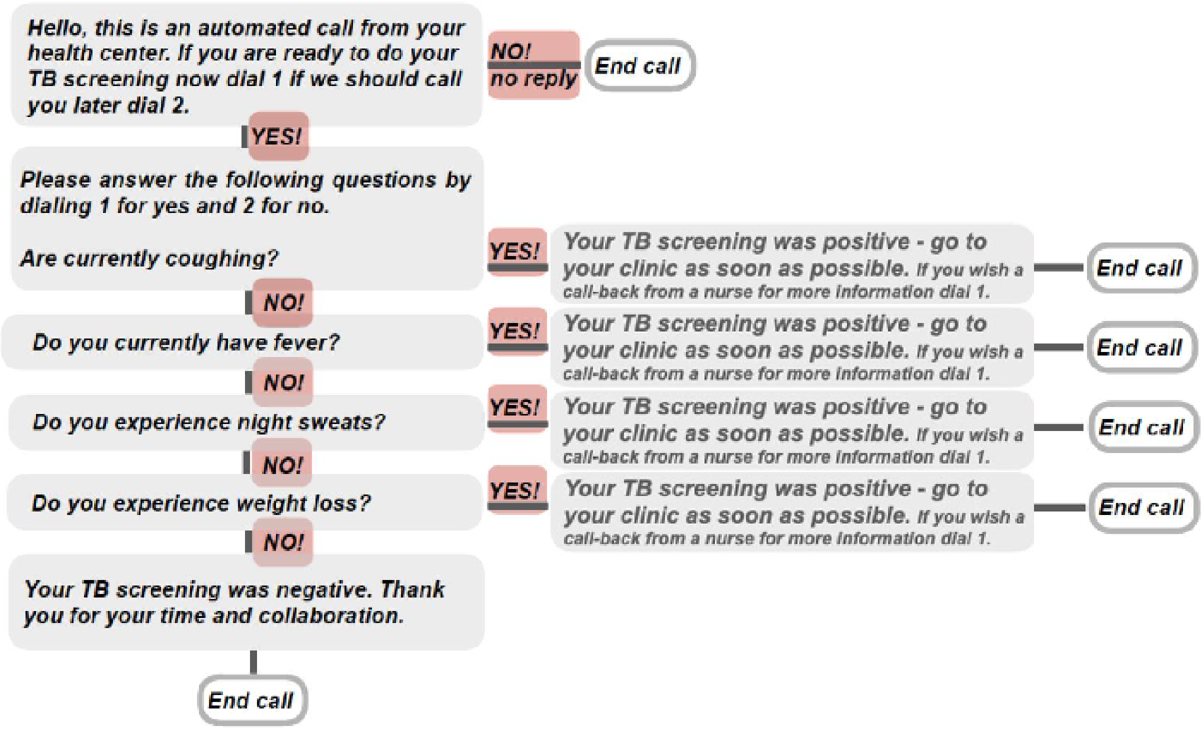
Hello, this is an automated call from your health center. If you are ready to do your TB screening now dial 1 if we should call you later dial 2.
NO! no reply
End call
YES!
Please answer the following questions by dialing 1 for yes and 2 for no.
Are currently coughing?
YES!
Your TB screening was positive - go to your clinic as soon as possible. If you wish a call-back from a nurse for more information dial 1.
End call
NO!
Do you currently have fever?
YES!
Your TB screening was positive - go to your clinic as soon as possible. If you wish a call-back from a nurse for more information dial 1.
End call
NO!
Do you experience night sweats?
YES!
Your TB screening was positive - go to your clinic as soon as possible. If you wish a call-back from a nurse for more information dial 1.
End call
NO!
Do you experience weight loss?
YES!
Your TB screening was positive - go to your clinic as soon as possible. If you wish a call-back from a nurse for more information dial 1.
End call
NO!
Your TB screening was negative. Thank you for your time and collaboration.
End call
